# Distribution of Recombination Hotspots in the Human Genome – A Comparison of Computer Simulations with Real Data

**DOI:** 10.1371/journal.pone.0065272

**Published:** 2013-06-11

**Authors:** Dorota Mackiewicz, Paulo Murilo Castro de Oliveira, Suzana Moss de Oliveira, Stanisław Cebrat

**Affiliations:** 1 Department of Genomics, Faculty of Biotechnology, University of Wrocław, Wrocław, Poland; 2 Instituto de Física, Universidade Federal Fluminense, Niterói, Rio de Janeiro, Brazil; 3 National Institute of Science and Technology: Complex Systems, Rio de Janeiro, Brazil; Tulane University Health Sciences Center, United States of America

## Abstract

Recombination is the main cause of genetic diversity. Thus, errors in this process can lead to chromosomal abnormalities. Recombination events are confined to narrow chromosome regions called hotspots in which characteristic DNA motifs are found. Genomic analyses have shown that both recombination hotspots and DNA motifs are distributed unevenly along human chromosomes and are much more frequent in the subtelomeric regions of chromosomes than in their central parts. Clusters of motifs roughly follow the distribution of recombination hotspots whereas single motifs show a negative correlation with the hotspot distribution. To model the phenomena related to recombination, we carried out computer Monte Carlo simulations of genome evolution. Computer simulations generated uneven distribution of hotspots with their domination in the subtelomeric regions of chromosomes. They also revealed that purifying selection eliminating defective alleles is strong enough to cause such hotspot distribution. After sufficiently long time of simulations, the structure of chromosomes reached a dynamic equilibrium, in which number and global distribution of both hotspots and defective alleles remained statistically unchanged, while their precise positions were shifted. This resembles the dynamic structure of human and chimpanzee genomes, where hotspots change their exact locations but the global distributions of recombination events are very similar.

## Introduction

Analyses of meiotic recombination in the human genome revealed uneven distribution of recombination events along chromosomes. This phenomenon has been observed at different genomic scales. At the megabase scale, the average recombination rate is higher in the subtelomeric regions than in the middle part of chromosomes [1]–[4]. This uneven distribution of recombination events has also been observed in other eukaryotic genomes [3], [5]–[9], suggesting its universal character. At the finer scale of the human chromosomes, recombination events tend to happen in narrow spans that are called recombination hotspots [10], [11]. They are usually 1–2 kb long regions where crossing over occurs much more frequently than in surrounding sequences. The distribution of these regions can differ among individuals, causing inherited patterns of recombination rate variation, which suggests that the pattern of recombination rate evolves [12]–[14]. The hotspot distribution is positively correlated with GC content and repetitive element distribution, whereas it shows a negative correlation with the gene density [15]. However, none of these relationships can reliably be used to predict the hotspot locations precisely [16]. In accordance with the large scale distribution of recombination events, the hotspots locate more frequently in the subtelomeric regions of chromosomes than in their central parts and occur preferentially near genes, though they avoid transcribed regions. Moreover, the hotspots in terminal parts of chromosomes are more active than in other regions [15].

Analyses based on the genetic variation identified a few short DNA motifs overrepresented in human hotspots [15]. Further research suggested that the partially degenerated 13-mer motif CCNCCNTNNCCNC can play an important role in the hotspot activity [17]. Recently, it has been shown that the zinc finger protein called PRDM9 can bind to this motif and initiate recombination at its location [18]–[20]. However, recombinational activity was also observed in hotspots that do not have such motif [20]. Moreover, the degenerated motif was found only in approximately 40% of hotspots and was also present in regions that do not show any hotspot activity [17].

The comparison of hotspot locations between human and chimpanzee has shown that they are not conserved despite almost 99% identity of these species at the level of genomic sequence [9], [21], [22]. In addition, up to now, there is no evidence for the presence of a sequence motif typical of chimpanzee hotspots [9], whereas the analysis of the recombination landscapes among African Americans identified a novel 17-base pair motif [23]. The majority of data indicate that the different usage of hotspots in closely related species and also within an individual of a given species is connected with variations of the zinc finger domain in PRDM9 [14], [24]. However, it is still unclear how PRDM9 binds to DNA or if it needs other components for its activity [25]. Therefore, a role of other factors in the location and activity of recombination hotspots cannot be excluded. Chromatin configuration could be one of them [22], [26], as for example in yeast [11], [27].

In contrast to the short lifespan of hotspot locations, the distribution of recombination rates on the global scale, i.e. subtelomeric versus central part of chromosomes, is rather conserved [3], [4], [9]. This suggests that factors influencing the global recombination rate operate independently of the regulatory systems for the location and activity of individual hotspots [16].

The problem of recombination hotspots has also been a subject of theoretical studies [28]–[31]. Most of them focused on the explanation of hotspot survival in the context of the biased transmission caused by gene conversion. This problem is commonly known as the “recombination hotspot paradox” [28]. Attempts to solve it have mainly assumed selection favouring recombination as the process ensuring correct segregation of chromosomes during meiosis. However, this reasoning did not explain all aspects of recombination. Recently, Ubeda and Wilkins [32] have offered a new model that, according to the authors, includes all available empirical data related to recombination. This model assumes co-evolution between motifs and proteins that recognize them. It leads to dynamic equilibrium in the disappearance of hotspots and their generation in new places.

However, this model did not include other genetic information such as genes that code different functions not involved directly in recombination. In consequence, this model did not consider any direct selection for the generation of new gene combinations, which is one of the most important effects of meiosis. They also did not try to explain the commonly observed global distribution of hotspots and recombination events, i.e. their higher frequency at subtelomeric regions and lower in the middle of chromosomes. It is interesting to ask which selection force maintains this large-scale distribution of recombination events and simultaneously allows for very high frequency of hotspot relocation. Is it possible that such distribution could be self-organized if spatial organization of genetic information in the recombining chromosomes is present?

We assumed that the “hotspot paradox” is solved and it is possible to reach an equilibrium in appearance and disappearance of hotspots [32]. Therefore, we elaborated a Monte Carlo model of eukaryotic chromosome evolution that allows self-organization of spatial distribution of both recombination events and genes responsible for individuals’ adaptation and also their defective alleles subjected to negative selection. We obtained the global uneven distribution of hotspots with their transient nature without the assumption of any direct selection for hotspot occurrence or location in the virtual chromosome. The only selection force considered in the model was the diminished survival probability of diploid individuals (not gametes) with a higher fraction of defective alleles.

The results of the model were verified by comparison with the observed distribution of hotspots and recombination-associated DNA motifs along the human chromosomes. We showed that the distribution of motif clusters is correlated with the distribution of recombination rate rather than the distribution of single motifs.

## Materials and Methods

### Data on Recombination and Hotspot Distribution

The human genetic map and locations of recombination hotspots were downloaded from the HapMap web site [33], [34]. We used Phase II HapMap data that were estimated with methods described by McVean et al. [35] and Winckler et al. [22] from release 22 of the genetic map and release 21 of the recombination hotspot locations. The coordinates of hotspots that were consistent with the genome assemblies of hg17 were converted with Batch Coordinate Conversion (liftOver) software to the genome assemblies of hg18, at the website of UCSC Genome Bioinformatics Group (http://www.genome.ucsc.edu).

To present the distribution of hotspots along the human chromosomes, we applied a kind of DNA walk – detrended cumulative plots. To build the plot we used a virtual ‘walker’ moving along the chromosome (*x*-axis coordinate). When it encountered a hotspot (it could be any other declared sequence, see below) it summed them up from the beginning of the chromosome, calculated the expected number of hotspots in the visited part of the chromosome under the assumption that they were distributed evenly along the whole chromosome, and then it subtracted the expected number from the given one and put the result at the proper place in the plot (*y*-value corresponding to the *x*-axis coordinates). In such a plot, regions of chromosome with increasing *y*-values are overrepresented in hotspots whereas regions with decreasing *y*-values are underrepresented.

### Distribution of Motifs along Chromosomes

All analyses comparing recombination data and hotspot locations with the parameters of human chromosomes were based on the hg18 human genome assembly (NCBI build 36.1, March 2006). The sequences of chromosomes were downloaded from the University of California, Santa Cruz (UCSC Genome Browser; http://www.genome.ucsc.edu) [36]. Data for chimpanzee chromosomes were obtained from the panTro2 assembly (CHIMP2.1, Mar 2006). The downloaded sequences were searched for the degenerate 13 bp motif CCNCCNTNNCCNC, overrepresented in human hotspots [17], by using the fuzznuc program from the EMBOSS package (ftp://emboss.open-bio.org/pub/EMBOSS/) [37]. The distribution of the motif along human chromosomes was analysed both in the physical scale in nucleotides [bp] and in the genetic scale in centimorgans [cM].

We analysed the distribution of distances between found motifs by counting the distance from start to start between each pair of neighbouring motifs in physical and genetic scales. To test if the distribution of distances between motifs observed in human chromosomes is expected by chance, we randomized motif locations 100 times separately for each chromosome and calculated the distances between them. The minimum and maximum of these distances were used for comparison with the real data.

In addition, we studied the distribution of all found motifs as well as clusters of these motifs, which we grouped into: (i) consecutive motifs located only in one DNA (Watson or Crick) strand, (ii) consecutive motifs located in opposite DNA strands, and (iii) motifs separated from each other by a distance shorter than the average expected distance between motifs calculated for the chromosome. The neighbouring motifs located at a distance larger than the average were called unclustered. To compare distributions of motifs with hotspots in chromosomes, we correlated the number of these items found in corresponding fragments of the chromosome and calculated the Spearman’s rank correlation coefficient.

The analyses were done for all human autosomes but the results for chromosome 6 were presented as an example.

### The Model of Computer Simulations

**Figure 1:**
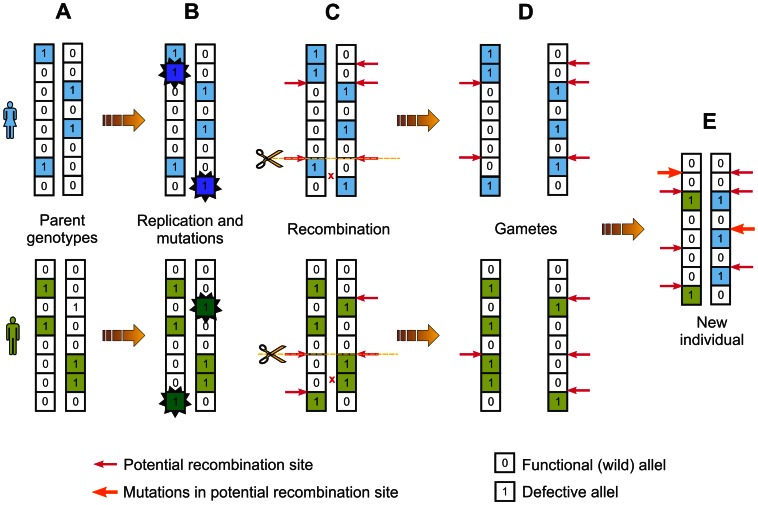
Stages of generation of a new individual in virtual genome evolution. **A**. Two diploid parental genomes participate in the generation of a new offspring. Their chromosomes, represented here by 8 bit strings, consisted of 2048 bits in computer simulations. Defective alleles are indicated by *1*, wild alleles by *0*. **B**. During the genome replication, a new mutation, marked by an asterisk, is introduced with probability *M* into the replicated chromosomes. **C**. During the formation of gametes, the new copies of bitstrings recombine with probability *C* at the intergenic site randomly chosen from all allowed crossover sites. A given position is considered as “allowed” if both corresponding positions in two bitstrings are hotspots (marked by the red arrow). **D**. The chromosomes after mutation and recombination create gametes. **E**. The haploid gametes of two partners form the diploid zygote. During this process, one intergenic site in each haplotype is randomly chosen and a new hotspot is generated (indicated by the orange arrow) if this site has no hotspot otherwise the already existing hotspot is eliminated.

To reproduce the phenomena associated with natural recombination processes we have developed a computer model based on the Monte Carlo (MC) method (the source code is available on request to PMC de Oliveira via e-mail: pmco@if.uff.br). In our simulations, each MC step starts with a fixed-sized population of *N* individuals (Fig. 1). Each individual is represented by its diploid genome composed of two homologous chromosomes (bitstrings). Two bits occupying the corresponding positions in the bitstrings (locus) represent alleles responsible for the same function. A bit set to *0* corresponds to the functional (wild) allele and a bit set to *1* corresponds to the defective version of an allele (Fig. 1A). All defective alleles are recessive, which means that both alleles at a given locus have to be defective to determine the deleterious phenotype of that locus.

At the beginning of simulations, all founder individuals have only *0* bits in both bitstrings so they are perfect. At each time step, all individuals surviving the previous step and newborns undergo selection. A random number in the interval *(0,1)* is tossed for each individual and compared with its own survival probability. If the random number exceeds the probability, the individual dies. The survival probability depends on the number of accumulated mutations, according to the rule: *x^(d+1)^*, where *x* is a number lower than *1* and *d* is the number of homozygous defective loci in the genome of the individual. The more homozygous defective loci are present in the genome of an individual the lower is the probability of its survival. Thus, the individual fitness is determined by the number of defective genes responsible for its adaptation. Notice that even an individual with a perfect genome dies with the probability *1-x*. The extra factor *x* introduced by adding *1* in the exponent prevents mutation-free individuals from living forever. All individuals eliminated by selection at a given MC step of simulation are replaced by newborns before the next MC step.

The genomes of newborns are constructed in the reproduction process mimicking meiosis, gamete production and fertilization (Fig. 1B–E). Two individuals are randomly drawn from the survivor pool as mating partners. We do not distinguish between male and female. The genome of each partner is replicated and *M* mutations on average are introduced into each copy of the bitstring in random positions (Fig. 1B). In order to determine the specific number of mutations introduced during the formation of a particular gamete, first we toss a random number between *0* and *2M*. Its integer part is the number of mutations performed, plus a further mutation with the probability equal to its fractional part. For example, if the tossed number is *1.6* we introduce one mutation, and then with probability *0.6* another mutation. The bit drawn for mutation changes its value from *0* to *1* or *vice versa*, which means that there reversions are allowed. Gamete formation is preceded by reciprocal recombination (crossover) between two copies of bitstrings with probability *C* on average at a position randomly chosen from the “allowed” positions for recombination between genes (Fig. 1C). The “allowed” position is when both of two recombining bitstrings possess a hotspot (a potential recombination site) at the corresponding positions. Such positions are also called double hotspots in contrast to single hotspots that do not match a corresponding hotspot in the homologous bitstring. Similarly to mutations, the number of crossovers is randomly taken from the interval between *0* and *2C*.

In the initial population, each founder individual has only a single randomly chosen position for recombination. Thus, in the whole population, the initial positions of recombination hotspots are evenly distributed along chromosomes. Therefore, it is possible in a few successive generations that no individual has an “allowed” position for recombination because there are no inherited hotspots is in the corresponding positions on homologous chromosomes. In such a situation, the position of recombination is randomly chosen and after the recombination both bitstrings keep hotspots there. This method of new hotspot generation takes places only at the very first steps of simulations when the number of hotspots in the genomes is low. Each newborn inherits hotspots from both parental bitstrings (Fig. 1D). In addition to the inherited hotspots, one mutation event per copy of chromosome introduces a new hotspot into the randomly chosen intergenic site or eliminates it if it already exists in the chosen site (Fig. 1E). Recombination at hotspots does not influence their distribution directly, but it can influence the number and distribution of hotspots in a single bitstring by reshuffling the existing ones in the two parental bitstrings. The haploid gametes of two partners fuse and form a diploid individual which undergoes selection in the next MC step. Selection is based exclusively on the genetic status of individuals, i.e. the number of homozygous defective loci.

## Results and Discussion

### Distribution of Hotspots and Motifs in the Human Genome

**Figure 2:**
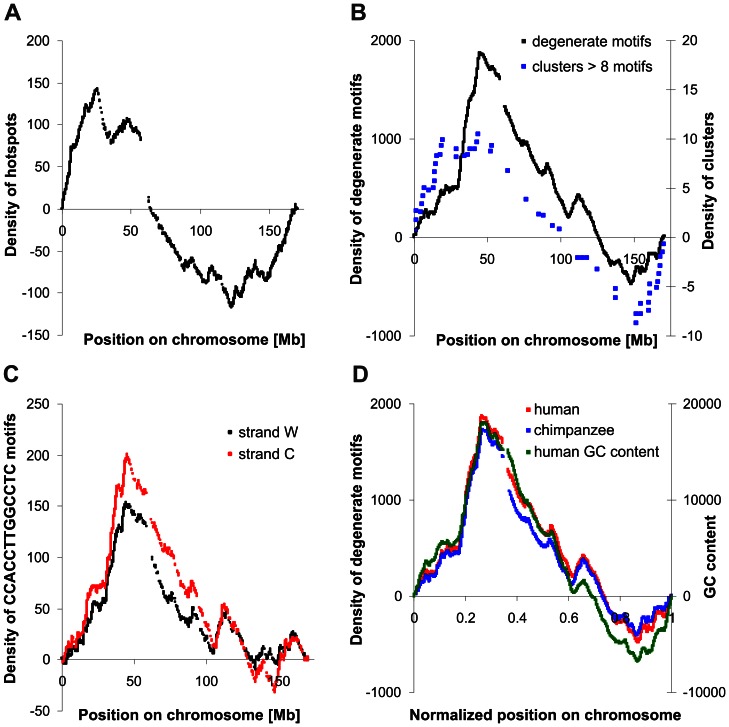
Detrended cumulative plots based on data for the human chromosome 6. **A**. Distribution of recombination hotspots along the chromosome. **B**. Distribution of the CCNCCNTNNCCNC (degenerate) motif and distribution of motif clusters along the chromosome. Data presented in this plot were obtained for clusters of eight or more motifs located on the same (Watson or Crick) DNA strand. **C**. Distribution of the specific CCACCTTGGCCTC motif along the chromosome for Watson and Crick strands separately. **D**. Comparison of the degenerate motif distribution along the human and chimpanzee chromosomes with the GC content for the human chromosome counted in 8 kb non-overlapping windows. The positions on the chromosome were normalized by division of real positions by the length of the human or chimpanzee chromosome 6, respectively. The increasing trends in the plots represent regions richer in hotspots or motifs than would be expected if they were evenly distributed in the chromosome, whereas the decreasing trends show the regions underrepresented in hotspots or motifs. The lack of points in the plot corresponds to the location of the centromere.

The uneven distribution of recombination events along human chromosomes has been observed in analyses of human family-based maps [2], [14] as well as in analyses of linkage disequilibrium (LD) patterns inferred from high-density single-nucleotide polymorphism (SNP) data [33], [34]. This uneven distribution is clearly visible in detrended cumulative plots [38]–[40]. Therefore, we applied this method for the analysis of distribution of hotspots and the degenerate motif CCNCCNTNNCCNC, which is overrepresented inside the human hotspots and is supposedly related to their activity [17]. These plots are presented in Fig. 2.

The distribution of recombination hotspots along all human autosomes shows that these short regions involved in recombination occur more frequently in the subtelomeric regions of chromosomes than in their centre (Fig. 2A). It corresponds to the distribution of recombination rate, which is also higher at the ends rather than in the middle of chromosomes [1]–[4]. It has been assumed that the position of centromere is responsible for the suppression of recombination in the central part of chromosomes [41], [42]. Moreover, it has been found that the proper segregation of chromosomes during meiosis requires at least one crossover per chromosome arm [43]. These findings suggest that recombination should have a symmetrical distribution around the centromere. However, analyses of distribution of recombination events on human chromosomes have shown that generally they are distributed symmetrically around the centre of chromosomes rather than the centromeres. The average distance between the physical centre and the genetic centre of human chromosomes (i.e. the point dividing the chromosome into two parts with the same total recombination probability) equals 5.2 Mb with standard deviation (SD) ±3.7 and it is significantly lower (Wilcoxon rank sum test, P<0.001) than the average distance measured between the physical centre and the centromere position (26.1±13.5 SD [Mb]). This suggests that the centromere is not directly responsible for the low recombination rate in the middle of chromosomes in humans. The distance between the intersection point of cumulative plots with the *x*-axis and the centre of chromosomes (13.5±13.3 SD [Mb]) is also much smaller (Wilcoxon rank sum test, P<0.001) than the distance from intersection points to centromeres (32.5±20.4 SD [Mb]). It also indicates the symmetric distribution of recombination events in chromosomes. These observations are in agreement with the results obtained by Jensen-Seamen et al. [3].

Because there is a relation between the occurrence of degenerate motif CCNCCNTNNCCNC and hotspots [19], [20], we checked if the distribution of this motif follows the non-random distribution of recombination events or hotspots along chromosomes. We assumed that if this motif is really related to recombination, its distribution should be similar to that of the recombination rate and hotspots. Our analyses confirm this expectation. In general, this motif is more frequent in the subtelomeric regions than in the middle part of chromosomes (Fig. 2B). It follows the distribution of recombination events and hotspots along chromosomes (Fig. 2A).

The studied motif consensus sequence was found in 14 917 copies in chromosome 6, while the most frequently represented sequence CCACCTTGGCCTC, belonging to the consensus, was found in 2 598 copies. We did not observe significant differences in the distribution of the CCACCTTGGCCTC motif as well as other sequences of the consensus (Fig. 2C). We also found a high co-linearity in the distribution of the degenerate CCNCCNTNNCCNC motif with the CCTCCCT motif, the first one that was observed in hotspots [15], and with the core motif CCTCCCTNNCCAC [17] (data no shown). The strong correlation between the distribution of motifs and hotspots suggests that motifs are involved in hotspot activity. Although they are not represented in all human hotspots, they dominate in regions where the recombination rate is very high. A similar trend in the motif distribution is visible for chromosome 6 of chimpanzee (Fig. 2D). Moreover, this motif is more overrepresented in the chimpanzee genome than in the human genome [9], [44]. It is an intriguing result because it has been shown that the 13 bp motif does not seem to act in determination of recombination positions in chimpanzee [9]. This raises a question why selection has conserved these motifs and their distribution in the genome. The assumption that this motif is not related to recombination in the chimpanzee genome may be premature.

On the other hand, motif distribution corresponds very well to the GC distribution along chromosomes: the higher the GC content, the higher the density of the motif is (Fig. 2D). One can suggest that the nucleotide composition rather than the recombination constraints is responsible for the 13 bp motif distribution. However, the probability of generating the motif even in the region rich in GC is very low. For example, the most frequently found CCACCTTGGCCTC motif in chromosome 6 is represented by 2 598 copies, whereas only a few copies could be expected if it is generated randomly in the corresponding nucleotide composition. The opposite situation, when many GC-rich motifs in a given chromosome region increase its GC content, is also impossible because the motifs are too short and too few in the global genomic scale. Thus, the correlation between the motif and GC content distribution is not driven just by the local nucleotide composition. It has to be connected with some other indirect phenomena.

### Analysis of Distances between Motifs

**Figure 3:**
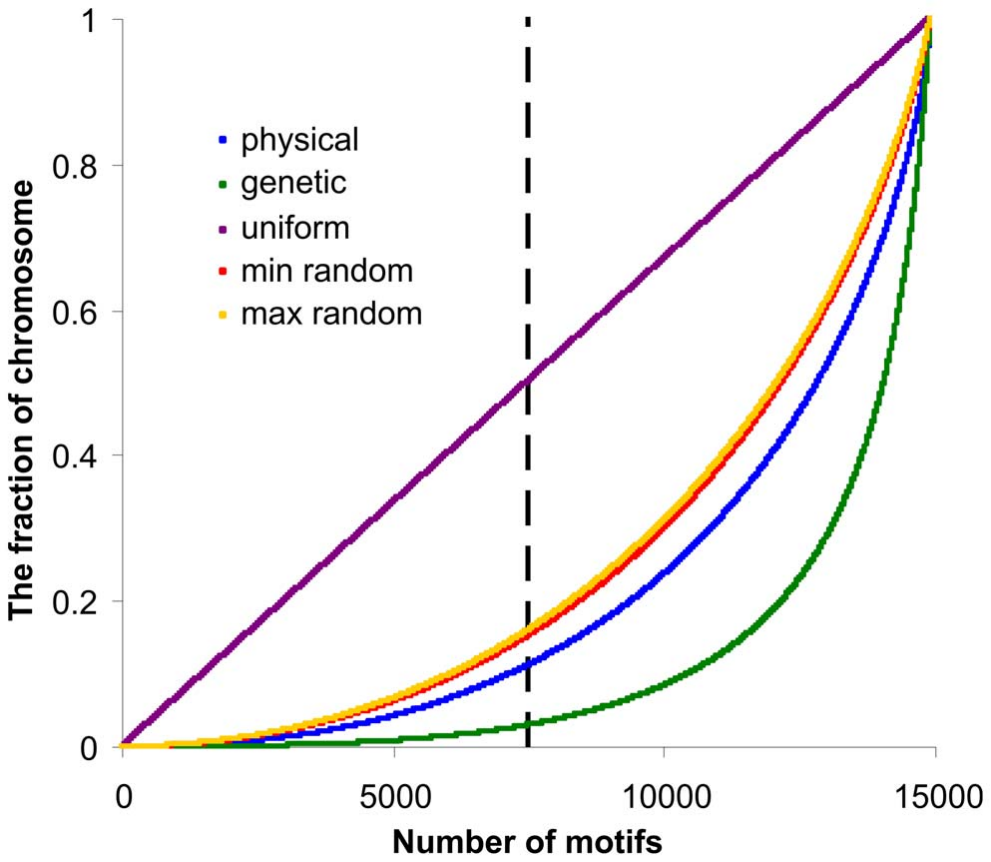
Analyses of distances between neighbouring motifs. To make a single plot, distances between motifs were presented as fractions of the whole chromosome, sorted ascending, and then the distances were cumulated from the shortest to the longest. Plots represent distributions of distances between degenerate 13-bp motifs for the human chromosome 6 in the genetic and physical scale. The same number of motifs distributed randomly or according to the uniform distribution are shown for comparison. In the case of random distribution, the minimum and maximum of distances between motifs locations are presented. Note that half of all distances analysed in this way (marked by the vertical dashed line) constitute 0.03, 0.11, 0.15, 0.5 of the chromosome length for the genetic and physical scales as well as for the random and uniform motif distributions, respectively. It means that motifs are the most clustered when analysed in the genetic scale.

To check more exactly how the DNA motif related to recombination is arranged in chromosomes, we calculated distances between neighbouring motifs measured in base pairs and in centimorgans (cM). The distances were sorted in ascending order and their lengths were cumulated (*y*-axis) from the shortest to the longest. The total length of each chromosome was normalized to 1. The number of cumulated distances between motifs is shown on the *x*-axis. Results of these analyses are shown in Fig. 3. The curve representing distances between evenly distributed motifs is a diagonal. There are two curves representing distances between randomly distributed motifs. They show the smallest and largest distances obtained from 100 independent randomized distributions of the same number of motifs. They roughly overlap in the scale in Fig. 3. The motifs are distributed unevenly on real chromosomes, because the curves are below the diagonal. They have also non-random distribution because the curves are below the corresponding curve for random distribution in both physical and genetic scales. The motifs have a tendency to appear in clusters, suggesting that they can co-occur at the same recombination hotspot, as it was reported by Myers et al. [17]. We found that this observation is true for all human autosomes. In chromosome 6, half of the distances between neighbouring motifs constitute only 11% of the chromosome length in the physical scale. Moreover, the motifs have their closest neighbour at a distance not larger than 0.2 of the average distance between motifs. The clustering of motifs in the same hotspot is even more visible when distances between them are analysed in the genetic scale. In this case, half of the shortest distances between neighbouring motifs constitute less than 3% of the whole chromosome measured in cM (Fig. 3). Furthermore, spacers between motifs in some clusters are very short and equal in length (e.g., 38, 58 or 134 bp) or are the multiple of a basic unit (e.g., of the length 16, 32, 48, 64 bp). The identity of multiplied sequences located between motifs is very high, suggesting that they are repetitions of a single sequence. In such clusters all motifs are also repeats of exactly the same sequence and they are located on the same DNA strand (Watson or Crick). This indicates that the motifs belong to repeated sequences. The relationship between motifs and repeated sequences is also confirmed by the common presence of these motifs within a specific mobile element, the long terminal repeat of a retrovirus-like retrotransposon of an inactive repeat element family (THE1 elements) or currently active element families, including Alu or LINE2 elements. It was observed that such surroundings increase hotspot activity in the human genome [15], [17]. However, this phenomenon imposes a question why hotspots and motifs are preferably located within repeated sequences, giving the potential for double-strand breaks in repeat DNA, which can lead to pathological rearrangements. It is possible that such a location helps in creation of new recombination sites [45]. However, it is worth noting that such organization indicates simultaneously that motifs arise by amplifications or translocations rather than by simple point mutations.

**Figure 4:**
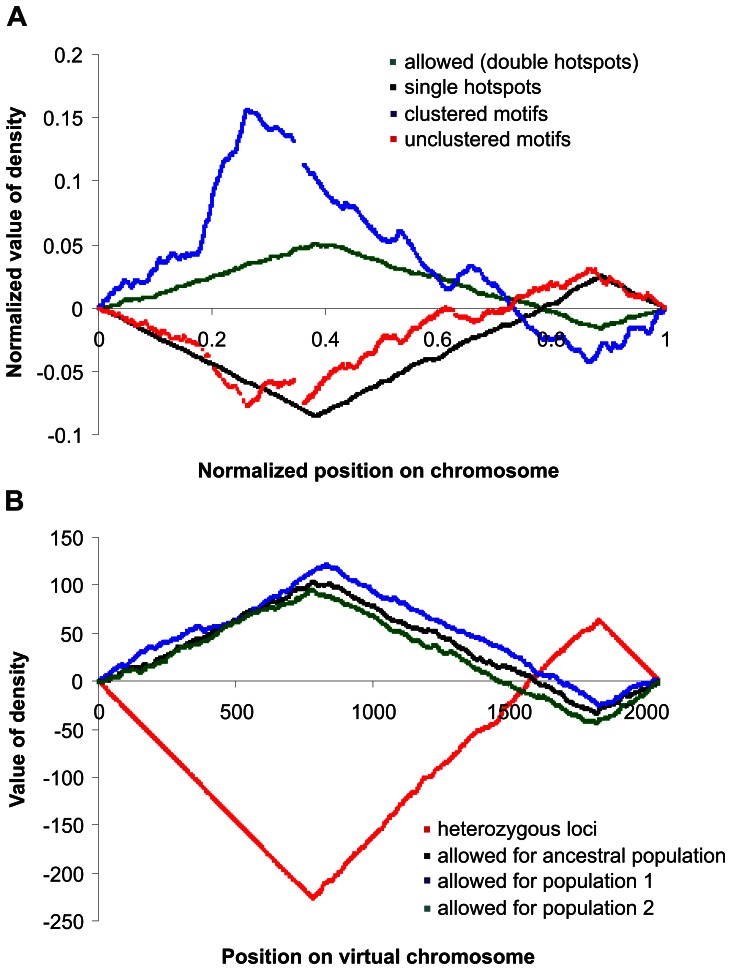
Detrended cumulative plots for real data and results from computer simulations. **A**. Comparison of hotspot distribution in a virtual chromosome with motif distribution in the human chromosome 6. Positions “allowed” for recombination (double hotspots) are sites in the virtual chromosome where recombination can occur whereas single hotspots are sites where recombination is prohibited. Motifs were considered clustered when they were located in the chromosome closer to each other than at the average distance between all motifs. In contrast to that, the unclustered motifs were separated by larger than average distances. **B**. Distribution of heterozygous loci (defective alleles) and double hotspots (“allowed”) for the ancestral and two descendant populations, which evolved independently from the ancestral one for the next 100 000 MC steps of simulations. Note that in the regions of chromosomes with a lower recombination rate, a higher fraction of heterozygous positions of genes is observed.

Since there are no simple, direct relations between motif occurrence and frequency of recombination events, we compared the distribution of single motifs with the distribution of motifs grouped in clusters along the chromosomes. We divided all motifs into two sets: (i) clustered motifs - that had the closest neighbour at a distance no larger than the average distance between motifs, and (ii) unclustered motifs - that had the closest neighbour further than the average distance. The distribution of clustered motifs resembles the distribution of hotspots whereas the distribution of unclustered motifs shows just a reciprocal trend (Fig. 4A). The distribution of clustered motifs fits better to the hotspot distribution than the distribution of single motifs. These concordant trends are confirmed for the distribution of clusters composed of eight or more motifs in the case of chromosome 6 (Fig. 2B). The Spearman’s rank correlation coefficient between distribution of hotspots and motif clusters is larger (r = 0.6; p = 0.02) than the coefficient for all motifs (r = 0.5, p = 0.07). The analyses suggest that only motifs in clusters are involved in recombination events. The increase in the number of targets for recombination-related proteins could promote the recombination activity in chromosomes. The same phenomenon has been observed for another DNA consensus involved in DNA metabolism: boxes that bind DnaA proteins involved in the initiation of prokaryotic chromosome replication [46]. There are some clusters of DnaA boxes in the vicinity of the origin of replication and many other unclustered boxes dispersed along the whole chromosome [47]. It is assumed that single DnaA boxes act as a kind of buffer titrating DnaA protein in the cell [48]. In the case of recombination, it is possible that clusters of motifs are responsible for initiation of recombination events and single, unclustered motifs can be used as a buffer for binding PRDM9 protein in the inactive form or that they can be involved in the formation of synapses, alignments of homologous chromosomes during the early stages of meiosis.

### Distribution of Recombination Hotspots in Virtual Genomes

We have developed a computer model based on the Monte Carlo method to reproduce the phenomena associated with natural recombination processes. We implemented to this model the spatial distribution of both recombination events and genes responsible for adaptation of individuals. Probabilities of recombination at given positions are inherited, though they can be changed by mutations. This model does not assume any direct selection for the hotspot occurrence or location in the chromosome. The only applied selection is against defective alleles. In our model, the distribution of recombination events can evolve, though the selection does not recognize the recombination hotspots themselves but only phenotypic deleterious loci.

The simulated population had the fixed size *N* = 500 individuals. The diploid genome of each individual was represented by two bitstrings of the length *L* = 2048 bits (genes). The bitstrings corresponded to homologous chromosomes and each of them can also be considered a haplotype. The selection force was *x* = 0.85. Note that *x* = 1 corresponds to the simulation without selection. The average mutation rate per bitstring per generation was *M* = 1 and the average recombination rate per bitstring per meiosis was *C* = 1. The mutation rate assumes Azbel’s calculations that the optimal rate of mutations should be around one mutation per genome per generation, independently of the size of the genome [49]. Many experimental results have shown that at least the order of this estimation is correct [50]. Parameters of the simulations were chosen on the basis of the previous analyses where the distribution of recombination events without the possibility of self-organization was studied [38]–[40], [51].

According to the applied parameters the number of potential recombination spots was 2047 (i.e. all intergenic positions) in each of two recombining bitstrings. Note that a recombination event was possible only when two recombining bitstrings possessed a hotspot at corresponding intergenic positions (Fig. 1). Such positions were called “allowed” positions or “double hotspot positions”. Thus, recombination did not happen if none of the corresponding intergenic positions in two recombining bitstrings had a hotspot or only one of these positions had it. The latter positions were called “single hotspot positions”. Both mutations of genes and hotspot generation were reversible. Gene state could change between the two values *0* and *1*, whereas the intergenic position could become a recombination hotspot or lose this property. We have assumed that the applied appearance and disappearance mechanism of hotspots caused by the mutational process well reflects natural phenomena observed in real genomes, where the loss of individual hotspots by biased gene conversion is balanced by a selective pressure creating novel target sequences for new variants of PRDM9 protein [19], [44].

**Figure 5:**
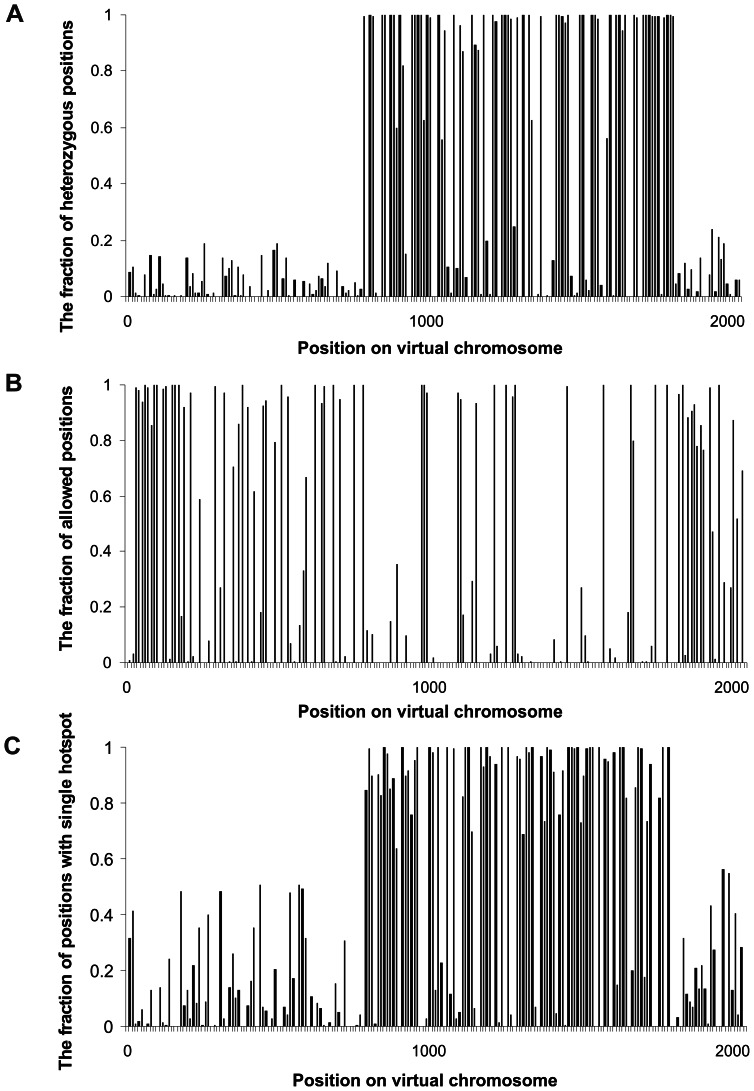
Genetic structure of the virtual chromosome after 3 million MC steps of simulations. **A**. Distribution of defective alleles (heterozygous positions). **B**. Distribution of double hotspots which allowed for recombination. **C**. Distribution of single hotspots in which recombination was impossible. Data for all plots were decimated to show more clearly the distribution of hotspots or defective alleles.

The results of simulations after 3 million MC steps are presented in Fig. 5. After that time, populations were in the steady state, i.e. the total number of hotspots and defective genes did not change significantly after the next 100 000 MC steps of simulations. One could expect that with the introduced reversions and without selection, the fraction of defective genes (bits set to *1*) and fraction of intergenic sequences occupied by hotspots should be 0.5. Nevertheless, in our simulations, the selection for defective genes was strong and the probability of survival depended directly on the number of defective loci in the individual’s genome (i.e. homozygous “*11*” loci). That is why the fraction of defective alleles (bits set to 1) was 0.32, instead of the expected 0.5, mainly in the heterozygous state. The fraction of homozygous defective loci was negligible and equalled 0.0003, which indicates the high effectiveness of selection. The distribution of heterozygous loci along chromosomes was not uniform (Fig. 5A). Most of them (94% of all heterozygous loci) were located in the middle part of the chromosome, between 783 and 1819 positions (Table 1).

**Table 1:** Distribution of hotspots and defective alleles in different regions of the virtual chromosomes after 3 million MC steps.

The observed number of:	The range of positions on virtual chromosome	
	1–783 and 1819–2047 (1012 loci)	784–1818 (1036 loci)
defective alleles	38	623
all hotspots	1049	972
double hotspots	469	201
single hotspots	111	570
positions without hotspots	432	264

Positions 1–783 and 1819–2047 are regions on the chromosome where the detrended cumulative plot for “allowed” positions (double hotspots) shows the increasing trend whereas the trend is decreasing in the region 784–1818 (see Fig. 4A). Since the fraction of homozygous defective loci is of the order of 0.0003, almost all defective alleles shown in the table are at the heterozygous positions. Double hotspots correspond to positions where recombination can occur, whereas at single hotspot positions recombination cannot happen.

This uneven distribution of defective loci (Fig. 5A) is clearly related to the distribution of hotspots (Fig. 5B,C). The expected number of hotspots in the diploid genome without any effect of selection was 2 047, i.e. half of all possible locations. The observed number of hotspots equalled 2 021 and seems to correspond to the expected one (99% of the expected value). Nevertheless, their distribution is completely non-random. One could expect that with the frequency of hotspots *p* (close to 0.5), fraction of positions with double (“allowed”) hotspots should be equal to *p* ^2^, 2*p*(1-*p*) for single hotspot positions and (1-*p*)^2^ for positions without any hotspot (see the expected numbers in Table 2). In fact, most of the hotspots were sequestered in “allowed” positions enabling recombination, whereas single positions were underrepresented. Furthermore, the “allowed” recombination sites were located mainly at the ends of chromosomes (Fig. 5B), whereas single positions were located preferentially in the central part (Fig. 5C). Interestingly, these trends are similar to the data obtained for human chromosomes for clustered and unclustered hotspot motifs (Fig. 4A). The probability to maintain “allowed” positions was higher at the ends of chromosomes, similarly to the location of motif clusters. It should be emphasized that hotspots (recombination sites) implemented in the computer model were initially distributed evenly along the chromosome and their generation and elimination were also random during the whole simulations. Therefore, only selection against defective alleles can be considered as the cause of the uneven hotspot distribution. This selection had a tendency to place groups of linked genes in the centre of the chromosome, especially in populations with a small effective size [38]–[40], [51]. These groups of genes consisted mainly of heterozygous loci that could complement their defects with other corresponding haplotypes present in the population. Therefore, when these groups became established by selection, any recombinations breaking these haplotypes had a deleterious effect and were selected against [38], [39]. Such groups of linked genes were not formed at the ends of chromosomes where purifying selection associated with a high recombination rate dominated. Such a preferential placement of the linked genes in the centre of chromosome suppresses recombination in this region that may influence the global distribution of recombination hotspots observed in human chromosomes. This is in agreement with the view that humans have evolved in relatively small populations [39], [52], in which the formation of linked genes is preferred [38]–[40], [51]. If we consider this problem from the point of evolutionary costs, it seems that avoiding recombination in a region of linked genes is more effective than elimination of recombinants from the population [38], [53]. Interestingly, the majority of hotspots detected by sperm typing (i.e. before selection) were also detected by indirect inference from linkage disequilibrium patterns [15]. This may suggest that some positions on chromosomes chosen for recombination are already subjected to selection.

**Table 2:** Comparison of simulated data after 3 million MC steps with expected results for hotspots.

Hotspot number	Double	Single	Positions without hotspots
observed	670	681	696
expected	499	1023	525

The total number of observed hotspots is 2021.

### Simulation of Evolution of Ancestral and Descendant Populations

To check if the location of defective genes and “allowed” recombination position are in a dynamic state, we prolonged simulations beyond 3 million MC steps. The population after this time was called the ancestral population and was used as the initial population for the next two parallel simulations with different random seeds. In this way we obtained two descendant independently evolving populations: population 1 and population 2. Data from the two parallel populations were collected after the next 10 000 and 100 000 MC steps. The results after the next 100 000 MC steps for the two descendant populations and for the ancestral population are presented in Fig. 4B. The global structure of the chromosome was not changed after the next 100 000 MC steps. Other global parameters, such as the total number of defective alleles, number of positions with a single hotspot, and number of positions without hotspots, did not change significantly, either. However, detailed analysis of the descendant populations showed that specific positions of hotspots did change. For each of the analysed populations, we assumed that the hotspot was fixed at a given position if at least 95% of individuals in the population possessed double hotspots at this position. In the ancestral population, 443 such positions were found. After 10 000 MC steps only half of them were found at the same position in the descendant populations, while after further 100 000 MC steps, the distribution of hotspots in the genomes was independent of their distribution in the ancestral genomes (Table 3). Only 28 of 443 (6%) positions where recombination was “allowed” were shared by the ancestral and both descending populations. In contrast to that, positions of single hotspots were more conserved. Almost 31% of such hotspots were shared by all three populations.

**Table 3:** The common number of “allowed” (double) and single hotspot positions between the ancestral and descendant populations.

Time of simulation after 3 M MCs	Population 1		Population 2		All populations	
	double	single	double	single	double	single
+10 000 MCs	238	383	205	378	109	247
+100 000 MCs	122	313	118	319	28	172

The number of double and single hotspot positions in the ancestral population was 443 and 562, respectively. The last column includes the number of hotspot positions common for all three populations, ancestral and the two descendant populations.

Because the evolution of recombination properties of chromosomes is related to the distribution of defective genes along them, we also checked how heterozygous positions were changed in the simulated populations (Table 4). We assumed that positions at which 95% of individuals are heterozygous are the fixed heterozygous position. All those highly heterozygous positions were found in the central region of chromosomes. The total number of heterozygous positions in the ancestral population was 530. In contrast to the 6% of double hotspot sites (Table 3), still about 25% of these heterozygous positions were preserved in the ancestral and descendant populations after the next 100 000 MC steps (Table 4). Generally, the common heterozygous positions disappeared in a way more similar to the single rather than double hotspots.

**Table 4:** The common number of defective alleles at heterozygous positions between the ancestral and descendant populations.

Time of simulation after 3 M MCs	Population 1	Population 2	All populations
+10 000 MCs	328	337	209
+100 000 MCs	265	261	132

The total number of heterozygous positions in the ancestral population was 530. The last column includes the number of heterozygous positions common for all three populations, ancestral and the two descendant populations.

The obtained results indicate that the distribution of recombination hotspots and defective alleles co-evolve. Clusters of linked genes complementing other alleles from corresponding haplotypes in the diploid genome have a tendency to locate in the central part of the chromosome. On the other hand, purifying selection eliminates defective alleles by recombinations in the subtelomeric regions, which prevents cluster formation. That is why the recombination rate is higher at the chromosome ends and most of the “allowed” hotspot positions are observed there. The divergence of the ancestral population into the parallel descendant lineages resembles the evolution of recombination patterns in human and chimpanzee genomes. The sizes of gene clusters remain conserved, similarly to the global recombination rate distribution, but the precise hotspot positions and the detailed evolution of genes inside the clusters stay in the dynamic state (Fig. 2D).

### The Influence of Model Modifications on the Obtained Results

In the above simulations, we have assumed that the probabilities of hotspot generation and their loss were equal. Similarly, defective mutations in genes occurred with the same probability as reversions. Adopting such conditions in a simulation without any selection, we can expect in the equilibrium that the fraction of defective genes should be equal to the fraction of wild alleles and the fraction of positions with hotspots should be equal to the fraction of positions without them. It should be emphasized that in our model, only defective genes were under direct negative selection whereas selection for hotspots could proceed only indirectly via the selection experienced by genes. In nature, reversion probability is much lower than the probability of gene destruction by mutation. That is why we checked a model without reversions. In such a model, defective genes could be generated by mutation but eliminated only by purifying selection. In addition, we studied a model when the hotspots could be neither generated nor eliminated. In this case, any changes in the number of recombination hotspots and their distribution were possible only due to recombination itself by reshuffling of hotspot positions in the parental haplotypes.

To study these assumptions, we performed simulations under parameters with equal forward and backward mutation rates as in the original model for the first 50 000 MC steps but next we continued two versions of simulations: (i) with switched off mutational changes of hotspots but left reversions for genes, and (ii) with both mutational changes of hotspots and gene reversions switched off. At the first glance, these versions produced distribution of defective alleles and “allowed” recombination positions resembling those in the model with reversions. Most of the defective genes in the heterozygous state were located in the central, complementing part of chromosomes where all hotspots stayed in the single state and decrease the probability of recombination. In the subtelomeric regions, a small number of defective genes were under purifying selection whereas hotspots existed mostly in the double state, allowing for recombinations. However, the genetic structure of the population was frozen because the number of different haplotypes shrunk, leaving only a few dominating haplotypes. The precise position of hotspots did not change during further evolution, even if no reversions of defective genes happened and selection had to eliminate them to keep the population alive. This indicates that to obtain the observed transient nature of hotspots, it is necessary to assume both appearance and disappearance of hotspot positions.

One can conclude that the assumption imposed on the model and restricting positions for recombination to only homozygous is not natural. That is why we also performed series of simulations where a hotspot in the single state could also participate in recombination. Interestingly, some strange effects appeared in such simulations. Since the probability of formation and elimination of hotspots was equal, one can expect that half of all possible positions would be occupied by hotspots after the long lasting simulations. However, the characteristic uneven global distribution of defective alleles and recombination positions was also observed in these simulations. Only 114 out of 2 054 hotspots were in the single state after 2 million MC steps, while 1 023.5 such positions were expected. An overwhelming majority of hotspots were in the double state (970 observed vs. 515.3 expected) and dominated at the ends of chromosomes.

### Conclusions

Computer models of chromosome evolution have shown that selection against defective alleles in diploid genomes can drive the recombination structure of chromosomes, i.e. (i) the symmetrical distribution of recombination events around the middle of chromosome [38]–[40], [51], [53], (ii) higher recombination rate in subtelomeric regions, and (iii) highly dynamic generation of recombination hotspots with their very frequent relocation in the chromosome. The distribution of recombination events is related to the distribution of defective alleles along chromosomes. In the central part of chromosomes, alleles tend to form clusters of linked genes which can complement clusters of alleles from corresponding haplotypes. That is why their structure is more conserved, because recombination events breaking the clusters produce gametes with a lower chance to complement another one in the diploid individual. In these regions, higher heterozygosis is observed, whereas in subtelomeric regions, recombination is more frequent and purifying selection more effectively eliminates defective alleles. Previous simulations of chromosome evolution in diploid genomes, with gene number and recombination rate close to those found for human chromosomes, have shown that (i) gene clustering initiates in the central part of chromosomes and next spreads to the lateral parts [38], [40], [51], (ii) recombination events inside the centre are unfavourable and (iii) selection eliminates individuals formed from gametes after such events. It is important that those previous simulations were performed with recombination events randomly distributed along the chromosome. It was selection which eliminated individuals formed from improper gametes. Thus, the costs of evolution were high because selection operated at the level of individuals already living in the environment. In this paper we have shown that selection operating at the level of genes is a force strong enough to introduce bias into the random distribution of recombination events and drive the optimal recombination structure of chromosomes. Since the probability of forming gametes after recombination inside the gene cluster is lower, such distribution enhances the success of reproduction. The process is self-organized in the model, suggesting that the same mechanism can generate genetic structure in eukaryotic chromosomes. The evolution of distribution of recombination events along chromosomes could be considered as evolvability – the evolution of processes that enhance the evolution rate or lead to novel functions that help the organism to reproduce.

## References

[1] Payseur BA, Nachman MW (2000) Microsatellite variation and recombination rate in the human genome. Genetics 156: 1285–1298. 11063702 10.1093/genetics/156.3.1285 PMC1461344

[2] Kong A, Gudbjartsson DF, Sainz J, Jonsdottir GM, Gudjonsson SA, et al (2002) A high-resolution recombination map of the human genome. Nat Genet 31: 241–247. 12053178 10.1038/ng917

[3] Jensen-Seaman MI, Furey TS, Payseur BA, Lu Y, Roskin KM, et al (2004) Comparative recombination rates in the rat, mouse, and human genomes. Genome Res 14: 528–538. 15059993 10.1101/gr.1970304 PMC383296

[4] Serre D, Nadon R, Hudson TJ (2005) Large-scale recombination rate patterns are conserved among human populations. Genome Res 15: 1547–52. 16251464 10.1101/gr.4211905 PMC1310642

[5] Prachumwat A, DeVincentis L, Palopoli MF (2004) Intron size correlates positively with recombination rate in *Caenorhabditis elegans* . Genetics 166: 1585–1590. 15082572 10.1534/genetics.166.3.1585 PMC1470791

[6] Barton AB, Pekosz MR, Kurvathi RS, Kaback DB (2008) Meiotic recombination at the ends of chromosomes in *Saccharomyces cerevisiae* . Genetics 179: 1221–1235. 18562657 10.1534/genetics.107.083493 PMC2475728

[7] Groenen MAM, Wahlberg P, Foglio M, Cheng HH, Megens H-J, et al (2009) A high density SNP-based linkage map of the chicken genome reveals sequence features correlated with recombination rate. Genome Res 19: 510–519. 19088305 10.1101/gr.086538.108 PMC2661806

[8] Backström N, Forstmeier W, Schielzeth H, Mellenius H, Nam K, et al (2010) The recombination landscape of the zebra finch *Taeniopygia guttata* genome. Genome Res 20: 485–495. 20357052 10.1101/gr.101410.109 PMC2847751

[9] Auton A, Fledel-Alon A, Pfeifer S, Venn O, Ségurel L, et al (2012) A fine-scale chimpanzee genetic map from population sequencing. Science 336 193–198. 22422862 10.1126/science.1216872 PMC3532813

[10] Jeffreys AJ, Kauppi L, Neumann R (2001) Intensely punctate meiotic recombination in the class II region of the major histocompatibility complex. Nat Genet 29: 217–222. 11586303 10.1038/ng1001-217

[11] Petes TD (2001) Meiotic recombination hot spots and cold spots. Nat Rev Genet 2: 360–369. 11331902 10.1038/35072078

[12] Kong A, Barnard J, Gudbjartsson DF, Thorleifsson G, Jonsdottir G, et al (2004) Recombination rate and reproductive success in humans. Nat Genet 36: 1203–1206. 15467721 10.1038/ng1445

[13] Coop G, Wen X, Ober C, Pritchard JK, Przeworski M (2008) High-resolution mapping of crossovers reveals extensive variation in fine-scale recombination patterns among humans. Science 319: 1395–1398. 18239090 10.1126/science.1151851

[14] Kong A, Thorleifsson G, Gudbjartsson DF, Masson G, Sigurdsson A, et al (2010) Fine-scale recombination rate differences between sexes, populations and individuals. Nature 467: 1099–1103. 20981099 10.1038/nature09525

[15] Myers S, Bottolo L, Freeman C, McVean G, Donnelly P (2005) A fine-scale map of recombination rates and hotspots across the human genome. Science 310: 321–324. 16224025 10.1126/science.1117196

[16] Paigen K, Petkov P (2010) Mammalian recombination hot spots: properties, control and evolution. Nat Rev Genet 11: 221–233. 20168297 10.1038/nrg2712 PMC4389181

[17] Myers S, Freeman C, Auton A, Donnelly P, McVean G (2008) A common sequence motif associated with recombination hot spots and genome instability in humans. Nat Genet 40: 1124–1129. 19165926 10.1038/ng.213

[18] Jeffreys AJ, Neumann R (2009) The rise and fall of a human recombination hot spot. Nature Genet 41: 625–629. 19349985 10.1038/ng.346 PMC2678279

[19] Baudat F, Buard J, Grey C, Fledel-Alon A, Ober C, et al (2010) PRDM9 is a major determinant of meiotic recombination hotspots in humans and mice. Science 327: 836–840. 20044539 10.1126/science.1183439 PMC4295902

[20] Berg IL, Neumann R, Lam KW, Sarbajna S, Odenthal-Hesse L, et al (2010) PRDM9 variation strongly influences recombination hot-spot activity and meiotic instability in humans. Nature Genet 42: 859–863. 20818382 10.1038/ng.658 PMC3092422

[21] Ptak SE, Hinds DA, Koehler K, Nickel B, Patil N, et al (2005) Fine-scale recombination patterns differ between chimpanzees and humans. Nat Genet 37: 429–434. 15723063 10.1038/ng1529

[22] Winckler W, Myers SR, Richter DJ, Onofrio RC, McDonald GJ, et al (2005) Comparison of fine-scale recombination rates in humans and chimpanzees. Science 308: 107–111. 15705809 10.1126/science.1105322

[23] Hinch AG, Tandon A, Patterson N, Song Y, Rohland N, et al (2011) The landscape of recombination in African Americans. Nature 476: 170–175. 21775986 10.1038/nature10336 PMC3154982

[24] Berg IL, Neumann R, Sarbajna S, Odenthal-Hesse L, Butler NJ, et al (2011) Variants of the protein PRDM9 differentially regulate a set of human meiotic recombination hotspots highly active in African populations. Proc Natl Acad Sci U S A 108: 12378–12383. 21750151 10.1073/pnas.1109531108 PMC3145720

[25] Ségurel L, Leffler EM, Przeworski M (2011) The case of the fickle fingers: how the PRDM9 zinc finger protein specifies meiotic recombination hotspots in humans. PLoS Biol. 9: e1001211. 10.1371/journal.pbio.1001211 PMC3232208 22162947

[26] Wu TC, Lichten M (1994) Meiosis-induced double-strand break sites determined by yeast chromatin structure. Science 263: 515–518. 8290959 10.1126/science.8290959

[27] de Massy B (2003) Distribution of meiotic recombination sites. Trends Genet 19: 514–522. 12957545 10.1016/S0168-9525(03)00201-4

[28] Boulton A, Myers RS, Redfield RJ (1997) The hotspot conversion paradox and the evolution of meiotic recombination. Proc Natl Acad Sci U S A 94: 8058–8063. 9223314 10.1073/pnas.94.15.8058 PMC21556

[29] Pineda-Krch M, Redfield RJ (2005) Persistence and loss of meiotic recombination hotspots. Genetics 169: 2319–2333. 15687277 10.1534/genetics.104.034363 PMC1449581

[30] Peters AD (2008) A combination of *cis* and *trans* control can solve the hotspot conversion paradox. Genetics 178: 1579–1593. 18245829 10.1534/genetics.107.084061 PMC2278049

[31] Friberg U, Rice WR (2008) Cut thy neighbor: cyclic birth and death of recombination hotspots via genetic conflict. Genetics 179: 2229–2238. 18689896 10.1534/genetics.107.085563 PMC2516093

[32] Ubeda F, Wilkins JF (2011) The Red Queen theory of recombination hotspots. J Evol Biol 24: 541–553. 21159001 10.1111/j.1420-9101.2010.02187.x

[33] The International HapMap Consortium (2005) A haplotype map of the human genome. Nature 437: 1299–1320. 16255080 10.1038/nature04226 PMC1880871

[34] The International HapMap Consortium (2007) A second generation human haplotype map of over 3.1 million SNPs. Nature 449: 851–861. 17943122 10.1038/nature06258 PMC2689609

[35] McVean GA, Myers SR, Hunt S, Deloukas P, Bentley DR, et al (2004) The fine-scale structure of recombination rate variation in the human genome. Science 304: 581–584. 15105499 10.1126/science.1092500

[36] Kent WJ, Sugnet CW, Furey TS, Roskin KM, Pringle TH, et al (2002) The human genome browser at UCSC. Genome Res 12: 996–1006. 12045153 10.1101/gr.229102 PMC186604

[37] Rice P, Longden I, Bleasby A (2000) EMBOSS: the European Molecular Biology Open Software Suite. Trends Genet 16: 276–277. 10827456 10.1016/s0168-9525(00)02024-2

[38] Waga W, Mackiewicz D, Zawierta M, Cebrat S (2007) Sympatric speciation as intrinsic property of expanding populations. Theory Biosci 126: 53–59. 18087758 10.1007/s12064-007-0010-z

[39] Mackiewicz D, Zawierta M, Waga W, Cebrat S (2010) Genome analyses and modelling the relationships between coding density, recombination rate and chromosome length. J Theor Biol 267: 186–192. 20728453 10.1016/j.jtbi.2010.08.022

[40] de Oliveira PMC, Moss de Oliveira S (2011) The zipper effect: Why different positions along the chromosome suffer different selection pressures. Physica A 390: 492–498.

[41] Westphal T, Reuter G (2002) Recombinogenic effects of suppressors of position-effect variegation in Drosophila. Genetics 160: 609–621. 11861565 10.1093/genetics/160.2.609 PMC1461983

[42] Mahtani MM, Willard HF (1998) Physical and genetic mapping of the human X chromosome centromere: Repression of recombination. Genome Res 8: 100–110. 9477338 10.1101/gr.8.2.100

[43] Pardo-Manuel de Villena F, Sapienza C (2001) Recombination is proportional to the number of chromosome arms in mammals. Mamm Genome 12: 318–322. 11309665 10.1007/s003350020005

[44] Myers S, Bowden R, Tumian A, Bontrop RE, Freeman C, et al (2010) Drive against hotspot motifs in primates implicates the PRDM9 gene in meiotic recombination. Science 327: 876–879. 20044541 10.1126/science.1182363 PMC3828505

[45] McVean G (2010) What drives recombination hotspots to repeat DNA in humans? Philos Trans R Soc Lond B Biol Sci 365: 1213–1218. 20308096 10.1098/rstb.2009.0299 PMC2871820

[46] Messer W (2002) The bacterial replication initiator DnaA. DnaA and oriC, the bacterial mode to initiate DNA replication. FEMS Microbiol Rev 26: 355–374. 12413665 10.1111/j.1574-6976.2002.tb00620.x

[47] Mackiewicz P, Zakrzewska-Czerwińska J, Zawilak A, Dudek MR, Cebrat S (2004) Where does bacterial replication start? Rules for predicting the oriC region. Nucleic Acids Res 32: 3781–3791. 15258248 10.1093/nar/gkh699 PMC506792

[48] Ogawa T, Yamada Y, Kuroda T, Kishi T, Moriya S (2002) The datA locus predominantly contributes to the initiator titration mechanism in the control of replication initiation in *Escherichia coli* . Mol Microbiol 44: 1367–1375. 12068813 10.1046/j.1365-2958.2002.02969.x

[49] Azbel MY (1999) Universal unification of life, death, evolution, post-evolution and extinction. Physica A 273: 75–91.

[50] Drake JW, Charlesworth B, Charlesworth D, Crow JF (1998) Rates of spontaneous mutation. Genetics 148: 1667–1686. 9560386 10.1093/genetics/148.4.1667 PMC1460098

[51] Zawierta M, Waga W, Mackiewicz D, Biecek P, Cebrat S (2008) Phase transition in sexual reproduction and biological evolution. Int J Mod Phys C 19: 917–936.

[52] Liu H, Prugnolle F, Manica A, Balloux F (2006) A geographically explicit genetic model of worldwide human-settlement history. Am J Hum Genet 79: 230–237. 16826514 10.1086/505436 PMC1559480

[53] Cebrat S, Waga W, Stauffer S (2012) The role of haplotype complementation and purifying selection in the genome evolution. ACS 1: 1250041(1)–1250041 (24)..

